# Auditory training can improve working memory, attention, and communication in adverse conditions for adults with hearing loss

**DOI:** 10.3389/fpsyg.2015.00556

**Published:** 2015-05-28

**Authors:** Melanie A. Ferguson, Helen Henshaw

**Affiliations:** ^1^NIHR Nottingham Hearing Biomedical Research Unit, Otology and Hearing Group, Division of Clinical Neuroscience, School of Medicine, University of NottinghamNottingham, UK; ^2^Nottingham University Hospitals NHS TrustNottingham, UK

**Keywords:** auditory training, hearing loss, working memory, attention, communication, hearing aids, executive function, speech perception

## Abstract

Auditory training (AT) helps compensate for degradation in the auditory signal. A series of three high-quality training studies are discussed, which include, (i) a randomized controlled trial (RCT) of phoneme discrimination in quiet that trained adults with mild hearing loss (*n* = 44), (ii) a repeated measures study that trained phoneme discrimination in noise in hearing aid (HA) users (*n* = 30), and (iii) a double-blind RCT that directly trained working memory (WM) in HA users (*n* = 57). AT resulted in generalized improvements in measures of self-reported hearing, competing speech, and complex cognitive tasks that all index executive functions. This suggests that for AT related benefits, the development of complex cognitive skills may be more important than the refinement of sensory processing. Furthermore, outcome measures should be sensitive to the functional benefits of AT. For WM training, lack of far-transfer to untrained outcomes suggests no generalized benefits to real-world listening abilities. We propose that combined auditory-cognitive training approaches, where cognitive enhancement is embedded within auditory tasks, are most likely to offer generalized benefits to the real-world listening abilities of adults with hearing loss.

## Listening and Communication in Adverse Conditions

It is widely accepted that understanding speech in background noise is the most common problem for people with hearing loss (Vermiglio et al., 2012; Humes et al., 2013), as characterized by the typical statement “I can hear but I cannot understand what is being said.” In addition to a loss of hearing sensitivity, there may be additional deficits of temporal and spectral processing that contribute to listening difficulties (Hopkins and Moore, 2011). Furthermore, there is mounting evidence that non-sensory factors such as cognition, motivation, and context, play an important role in both listening to speech (one-way interaction process) and communication (bi-directional interaction; Kiessling et al., 2003; Pichora-Fuller and Singh, 2006; Rudner et al., 2011). This is particularly evident for older listeners (Gordon-Salant, 2014; Moore et al., 2014).

The role of cognition becomes more apparent when communicating in adverse conditions, such as when listening to speech in fluctuating background noise or competing talkers (Akeroyd, 2008; Humes and Dubno, 2010). Speech in noise performance is associated with cognition, and the role of cognition becomes increasingly important as the complexity of the listening task increases (Heinrich et al., 2015). For a listener to be able to understand a specific speech source amongst a background of other talkers, the auditory streams or sound sources need to be simultaneously attended to and monitored, and attention may need to be switched between them (Gatehouse and Noble, 2004; Shinn-Cunningham and Best, 2008). This requires the engagement of executive processes that regulate, control, and manage other cognitive processes, such as attention and working memory (WM; Chan et al., 2008).

## Cognition and the Clinical Management of People with Hearing Loss

The role of cognition has implications for the clinical management of people with hearing loss. Hearing aids (HAs) are the main intervention for people with hearing loss and have undergone significant advances in digital technology over the last two decades. Whilst satisfaction with HAs has improved since the 1990s (Kochkin, 2010), users often continue to encounter difficulties in challenging listening conditions (Johnson and Dillon, 2011). Early studies with HA users showed an association between behavioral and subjective HA outcomes and measures of cognitive skills (Gatehouse et al., 2003; Lunner, 2003). Furthermore, those with better cognitive skills were better able to take advantage of advanced signal processing strategies, such as fast-acting compression (Foo et al., 2007; Lunner and Sundewall-Thorén, 2007). Other processing strategies, such as noise reduction algorithms, have also been shown to reduce effortful listening and free up cognitive resources to be used for other tasks (Sarampalis et al., 2009).

When considering interventions to aid communication in people with hearing loss, HAs alone are not the only option. Other rehabilitation approaches include patient-centered education, counseling, and auditory perceptual training, which can help impaired listeners compensate for degradation in the auditory signal and improve communication (Sweetow and Sabes, 2006). This article focusses on developments in auditory training (AT), and more recently cognitive training, and how this may improve speech perception, cognition and ultimately, everyday communication in adults with hearing loss, offering a view to future research directions.

### Auditory Training

Auditory perceptual training can be described as teaching the brain to listen through active engagement with sounds, whereby listeners typically learn to make perceptual distinctions between sounds presented systematically (Schow and Nerbonne, 2006). Training on perceptual distinctions implies a primarily bottom-up approach to training whereby the individual actively listens to auditory stimuli (e.g., tones, phonemes, words) to improve listening and communication. This is reflected in the literature where traditionally, training studies have focussed primarily on the sensory refinement of auditory stimuli to improve speech perception (Fu et al., 2004; Stecker et al., 2006). But as Schow and Nerbonne’s (2006) definition suggests, the role of top–down cognitive processes is implicit in AT and subsequent learning. This has been demonstrated by training on non-auditory tasks, such as visual discrimination or visuospatial tasks, and auditory tasks with identical stimuli, resulting in learning in the auditory domain (Amitay et al., 2006). Such results imply that learning is mediated by top–down processes. Thus, AT may provide a means to improve both auditory and cognitive processes in people with hearing loss in order to improve listening and communication in everyday life (Pichora-Fuller and Levitt, 2012).

### Efficacy of Auditory Training

The turn of the last decade saw a proliferation of individualized, computer-based auditory training research. Basic research sought to better understand the underlying principles and biological mechanisms of AT in normally hearing listeners (e.g., Tremblay, 2007; de Boer and Thornton, 2008; Wright and Zhang, 2009; Song et al., 2011). In addition, translational research sought to establish the efficacy of AT to improve outcomes for people with hearing loss, including users of HAs and cochlear implants (for review, see Henshaw and Ferguson, 2013a). Efficacy of AT can be assessed by (i) improvements in performance for the trained task (on-task learning), (ii) improvements in performance on the untrained task (off-task, generalized, or transfer of learning), (iii) retention of learning for a period after training ceases, and (iv) adherence of the individual with training. This article concentrates on (i)–(iii). Motivations of individuals to participate in, engage with, and adhere to home-delivered training are discussed elsewhere (Henshaw et al., in review; Ferguson and Henshaw, in press).

Our recent systematic review on the efficacy of computer-based auditory training as a clinical intervention for adults with hearing loss summarized the evidence base between 1996 and 2011 and included 13 studies (Henshaw and Ferguson, 2013a). The review concluded that, where reported, on-task learning always occurred in those with mild-moderate hearing loss (whether HA users or not) for a range of training stimuli including phonemes, words, and sentences (e.g., Burk et al., 2006; Stecker et al., 2006; Sweetow and Sabes, 2006). The evidence for on-task learning in cochlear implants users generally followed this trend (e.g., Fu et al., 2004; Tyler et al., 2010; Oba et al., 2011) with the exception of Stacey et al. (2010). However, the evidence for generalization of learning to untrained measures was mixed. Although generalized improvements were shown for speech intelligibility (11/13 articles), self-report of communication (1/2), and cognition (1/1), the improvements were variable in that reported improvements were inconsistent across studies, and the magnitude of improvement was small and not robust. It was notable that all the studies had at least one outcome measure on speech intelligibility, yet different studies rarely used the same measure. Only two studies measured self-reported communication as a means to tap into perceived real-world benefits of training, and just one study measured cognition. The quality of the evidence for included studies was very-low to moderate. Reasons for this included failure to include a control group, and a lack of randomization, power calculation, and participant or tester blinding.

## Our Approach to Auditory Training

Following on from the systematic review, we sought to address many of the study quality limitations of the existing published evidence with a series of three high-quality auditory and cognitive training studies that aimed to assess benefits to speech perception, cognition, and self-reported communication in people with mild-moderate hearing loss. The study methods are outlined in **Table 1**. Outcome measures are shown in **Table 2**, and are described in more detail in the original articles.

**Table 1:** Study and participant characteristics.

Methods	Auditory training 1 Ferguson et al. (2014)	Auditory training 2 Henshaw and Ferguson (2014)	Working memory training Henshaw and Ferguson (2013b)
Study design	Randomized controlled trial	Repeated measures	Randomized controlled trial
Intervention	Phoneme discrimination in quiet 11 phoneme continua Phonomena/IHR STAR	Phoneme discrimination in multitalker babble 11 phoneme continua Phonomena/IHR Star	Verbal and Visuospatial working memory and storage tasks Cogmed RM
Intervention duration requested	360 min (6 h) across 4 weeks 15 min/day, 6 days/week (total = 24 sessions) Immediate Trained (IT): weeks 1–4 Delayed Training (DT): weeks 5–8	210 min (3.5 h) across 1 week (7 days), 2 × 15 min/day (total = 14 sessions)	Approximately 990 min (16.5 h) across 5 weeks, 35–45 min sessions/day, 5 days/week (total = 25 sessions)
Retention period	4 weeks post-training: IT: weeks 4–8 DT: weeks 8–12	None	6 months post-training
Control activity	None	None	Active control working memory tasks – span fixed at 3
Control period: duration	DT: T1–T2 = 4 weeks	T1–T2 = 1 week	T2–T3 = 5 weeks
Test–retest period	DT: T1–T2 = 4 weeks	T1–T2 = 1 week	T1–T2 = 1 week
n participants (n females)	44 (15)	30 (10)	57 (30)
Participants (source of recruitment)	Non-HA users, mild hearing loss (general practitioner)	Existing HA users, mild-moderate hearing loss (volunteer database)	Existing HA users, mild-moderate hearing loss (volunteer database)
Mean age in years (SD)	65.3 (5.7)	67.4 (7.1)	64.9 (6.0)
Mean BEA_0.5-4kHz_ (SD) dB HL	32.5 (6.0)	43.6 (13.6)	44.0 (13.8)

*IT, immediate trained; DT, delayed trained; HA, hearing aid; BEA, better-ear average; min, minute; T1–T2, time period between the first two test sessions to measure test–retest effects*.

**Table 2:** Summary of results for untrained tasks.

	Simple		Complex	
	Test	Results	Test	Results
**Auditory training 1: Ferguson et al. (2014) (*n* = 44)**	
Speech perception	Digit Triplet Test in steady speech-shaped noise^1^	NS within group for IT or DT (*p* > 0.05)	N/T	N/A
	Adaptive Sentence List in 8-Hz modulated noise^2^	NS within group effect for IT or DT (*p* > 0.05)	N/T	N/A
Cognition	Digit Span^3^	NS within group for IT or DT (*p* > 0.05)	Visual letter monitoring^4^ 1/s (fast) updating	All trained: *p* = 0.003, *d* = 0.50 DT control: NS (*p* > 0.05)
	Test of Everyday Attention^5^ – single attention task	NS within group for IT or DT (*p*> 0.05)	Test of Everyday Attention – dual task decrement	All trained: *p* = 0.001, *d* = 0.53 DT control: NS (*p* > 0.05)
Communication	Glasgow Hearing Aid Benefit Profile^6^: Television set to set to level for others 1:1 conversation in no background noise Conversation in a busy street or shop	NS within group for IT or DT (*p* > 0.05)	Glasgow Hearing Aid Benefit Profile: Having a conversation with several people in a group	All trained: *p* = 0.005, *d* = 0.68 DT control: NS (*p*> 0.05)
**Auditory training 2: Henshaw and Ferguson (2014) (*n* = 30)**	
Speech perception	N/A	N/A	Competing speech^7^	Trained period: *p* = 0.03, *d* = 0.47 Control period: NS (*p* > 0.05)
			Dual task of speech and memory^8^	Trained period: *p* = 0.001, *d* = 0.77 Control period: NS (*p* > 0.05)

*IT, immediate trained; DT, delayed Trained; NS, no significant effect; N/T, not tested; N/A, not applicable. ^1^Smits et al. (2004), ^2^Millward et al. (2011),^3^Wechsler (1997), ^4^Gatehouse et al. (2003), ^5^Robertson et al. (1996), ^6^Gatehouse (1999), ^7^Hazan et al. (2009), ^8^Howard et al. (2010)*.

Across all three studies, hearing loss was described by the better-ear pure-tone threshold averaged across octave frequencies 0.5–4 kHz as either mild (21–40 dB HL), or moderate (41–70 dB HL). Participants were aged 50–74 years old, and training was home-delivered either via loan laptops (AT studies) or via the internet (working memory training). Each study included a control period that allowed for the examination of procedural learning (test–retest) effects on outcomes (Mcarthur, 2007).

### Auditory Training Study 1: Training Improves Outcomes that Index Executive Function

The study was a randomized controlled trial, whereby a 4-week phoneme discrimination training program was performed for the Immediate Trained (IT) group at weeks 1–4, and a Delayed Trained (DT) group at weeks 5–8 provided a control comparison. Outcome measure assessments were obtained for the IT and DT groups at weeks 0, 4, and 8, and for the DT group at 12 weeks (Ferguson et al., 2014).

Results showed significant and robust on-task learning for all trained phoneme continua. The on-task learning and retention of on-task learning results were consistent with studies in the systematic review. However, from a clinical perspective the value of training as an intervention lies in the generalization of task-specific learning to functional benefits in real-world listening. A summary of the results from the untrained outcome measures is shown in **Table 2**, whereby tests and self-report questions were classified as complex if they indexed executive processes, and simple if they did not. Details of analysis using Multivariate Analysis of Variance is reported elsewhere (Ferguson et al., 2014). As we were also interested in the clinical effects of AT as an intervention, Cohen’s *d* is reported where effect size was interpreted as small (0.2), moderate (0.5), and large (0.8) (Cohen, 1988).

For the speech perception in noise tests that used energetic masking, there were no significant training-related improvements. For tests of cognition, there were no pre–post training improvements for the simple tasks, including simple-span WM measure (digit span) and the single attention test [Test of Everyday Attention (TEA) subtest 6] for either the intervention or control groups. However, for the complex tasks that indexed executive processes, there were significant pre–post training improvements shown for divided attention (TEA subtest 7) and the updating of WM (visual letter monitoring, VLM). For VLM there was a larger effect shown for the faster, more challenging presentation (one letter per second, *d* = 0.50) compared to the slower presentation (one letter every 2 s, *d* = 0.34).

For self-report of communication using the Glasgow Hearing Aid Benefit Profile (GHABP), there was a significant effect of training on the overall score for activity limitation (previously termed hearing disability) with a moderate effect size, suggesting real-world benefits were perceived by participants. A secondary analysis of the individual situations of the GHABP revealed an interesting insight in that no significant pre–post training improvements for the simple listening situations, such as ‘having a conversation with one other person when there is no background noise’ were shown. However, there was a significant effect of training for the most challenging listening situation ‘having a conversation with several people in a group.’ This requires the listener to constantly monitor the conversation, switch, and update attention (i.e., engage executive processes), whilst the other situations do not. These results were supported by qualitative analysis of open-ended questions and focus groups from participants who reported that the main benefits of the training were increased concentration and focus in everyday listening (Henshaw et al., in review). Across all measures where there were significant effects of training, these were retained 4 weeks post-training. Finally, in the participants where there were improvements in the GHABP measures, there was a significant correlation between self-report and divided attention (*r* = 0.79, *p* < 0.001), suggesting that improvements in self-report were not a ‘placebo’ effect of undertaking the training program.

These results suggest that outcome measures need to be appropriately complex and challenging to be sensitive to the effects of AT, and taken together, the value of AT to mediate cognitive skills may be more important than the improvement of sensory skills for communication in everyday life.

This led us to reconsider the non-significant speech perception results. Given that AT showed an improvement in the cognitive functions that index executive processes, we made the hypothesis that training-related improvements would be evident in informational masked speech perception tests (e.g., competing speech) that engage executive processes (Shinn-Cunningham, 2008), rather than the energetically masked speech tests that were included in this study, which primarily assess audibility. This was explored in study 2.

### Auditory Training Study 2: Training Improves Competing Speech and Dual-Task Performance

This study used a within-participant repeated measures design with an initial 1-week control period, followed by a 1-week training period (Henshaw and Ferguson, 2014). The training duration was 3.5 h, just over half that of the previous study, as the majority of the phoneme discrimination learning had taken place by this time. The modified co-ordinate response measure (MCRM) used a single female talker target and single male talker masker, presented simultaneously. The dual-task included a digit recall task (secondary), which flanked a word-in-noise repetition test (primary), presented at three signal-to-noise (SNR) levels (quiet, 0 dB and -4 dB).

Participants demonstrated significant on-task learning for the trained auditory task. Results for the untrained measures are shown in **Table 2**. For competing speech (MCRM), there was a significant pre–post training improvement with a moderate effect size and no improvement shown for the control (no-training) period. This confirmed our hypothesis and suggests that it is important to use appropriate speech measures that tap into the underpinning mechanisms of benefit provided by AT.

For the dual task, there was no effect of training for the easiest (quiet) or most difficult (-4 dB SNR) test conditions. However, there was a significant pre–post training improvement for the intermediate level of difficulty (0 dB SNR), with a large effect size. This suggests that the HA users in this study were better able to allocate their available cognitive resources between the speech and memory tasks post-training, and suggests that outcome measures need to be appropriately challenging in order to be sensitive to post-training improvements.

Given these results, we asked the question: “Could training cognition directly offer a more direct route to benefit for people with hearing loss?.”

## Working Memory Training for People With Hearing Loss

We used a WM training program (Cogmed RM) comprising verbal and visuospatial WM and memory storage tasks. Published studies of Cogmed RM have shown post-training improvements in untrained tasks of attention and self-report of cognitive function in younger and older adults (Brehmer et al., 2012), and improvements in sentence repetition for children with cochlear implants (Kronenberger et al., 2011).

### Working Memory Training: Training Results in Near-Transfer but not Far-Transfer of Learning

A registered clinical trial of 57 existing HA users with mild-moderate hearing loss assessed benefits to speech perception, self-reported communication, and cognition (for protocol, including outcome measures, see Henshaw and Ferguson, 2013b). In addition to assessing generalization to untrained tasks, we examined how far along the spectrum of near-transfer (e.g., outcome is close to the trained task) to far-transfer (e.g., untrained task in a different modality) any improvements occurred (Perkins and Salomon, 1992). Results (not yet published), showed near-transfer (i.e., improvements in an untrained WM task), but no far-transfer (e.g., speech perception) of training-related improvements, despite a longer training duration than for the AT studies. These results are consistent with the cognitive neuroscience literature, which shows that WM training can enhance WM tasks that share similar structural features (Thompson et al., 2013), however, training does not generalize to enhancement of the broader underlying cognitive constructs (Melby-Lervag and Hulme, 2013). It has been suggested that training-related improvements in trained WM tasks may be mediated by specific strategies, such as chunking or grouping (Dunning and Holmes, 2014), which may limit the broader applicability to benefit cognitive constructs underpinning successful communication for HA users.

## Auditory-Cognitive Training: Joined-up Listening and Thinking

Recent studies of an auditory-based cognitive training program that combines auditory perceptual training with increased memory demands (Brain Fitness; Posit Science) have demonstrated generalized improvements in non-trained tests of memory, attention, and speed of processing in older adults (Smith et al., 2009), in addition to improved neural timing and speech perception in noise (Anderson et al., 2013a,b). Similar results for a ‘hybrid’ training program comprising exercises of speech and cognition [Listening and Communication Enhancement (LACE), Sweetow and Sabes, 2006] trialed in mainly HA users, showed generalized improvements in speech in noise, auditory WM and speed of processing, in addition to improvements in self-report of communication difficulties. However, it is not clear from these studies which element of the training program is responsible for the transfer of learning.

## Future Directions

Following on from our own research and developments from the current literature, we propose that benefits of training for people with hearing loss in terms of improved speech understanding in adverse conditions may be best achieved if an integrated auditory-cognitive training approach is taken. This approach would serve to target the cognitive processes that underpin speech perception within a speech task, rather than training specific cognitive tasks that are far-removed from speech. One benefit of this approach is that the degree of transfer required to realize real-world benefit is substantially reduced. Furthermore, the nature of the speech task is more readily perceived as relevant to individuals in terms of their hearing difficulties, which is likely to aid motivation for adherence (Henshaw et al., in review).

Finally, a recent study has shown a dynamic relationship between WM capacity and speech recognition in the first 6 months of HA use with WM playing a greater role in speech perception initially, whereas after 6 months hearing sensitivity is more influential (Ng et al., 2014). Based on the Ease of Language Understanding model (Ronnberg et al., 2013), the authors suggest that as the unfamiliar processed phonological representations become more familiar with time, often referred to as acclimatization (Arlinger et al., 1996), there is a reduced requirement to use WM capacity for speech perception. However, the role of cognition in the acclimatization process is likely to extend beyond WM, and may call upon executive processes required for understanding speech. We are currently examining this in a longitudinal study of first-time HA users. Having identified which cognitive processes are important in acclimatization we aim to use a relevant auditory-cognitive training paradigm to minimize the difficulties faced in the early days of HA use.

## Author Contributions

MF and HH designed the studies. MF and HH analyzed and interpreted the data. MF wrote the manuscript. MF and HH contributed to manuscript revisions and critical discussions. Both authors approved the final version of the manuscript for publication. Both authors agree to be accountable for all aspects of the work and in ensuring that questions related to the accuracy or integrity of any part of the work are appropriately investigated and resolved.

## Conflict of Interest Statement

The authors declare that the research was conducted in the absence of any commercial or financial relationships that could be construed as a potential conflict of interest.

## References

[B1] Akeroyd M. A. (2008). Are individual differences in speech reception related to individual differences in cognitive ability? A survey of twenty experimental studies with normal and hearing-impaired adults. *Int. J. Audiol.* 47 S53–S71. 10.1080/14992020802301142 19012113

[B2] Amitay S. Irwin A. Moore D. R. (2006). Discrimination learning induced by training with identical stimuli. *Nat. Neurosci.* 9 1446–1448. 10.1038/nn1787 17028582

[B3] Anderson S. White-Schwoch T. Choi H. J. Kraus N. (2013a). Training changes processing of speech cues in older adults with hearing loss. *Front. Syst. Neurosci.* 7:97 10.3389/fnsys.2013.00097 PMC3842592 24348347

[B4] Anderson S. White-Schwoch T. Parbery-Clark A. Kraus N. (2013b). Reversal of age-related neural timing delays with training. *Proc. Natl. Acad. Sci. U.S.A.* 110 4357–4362. 23401541 10.1073/pnas.1213555110 PMC3600492

[B5] Arlinger S. Gatehouse S. Bentler R. Byrne D. Cox R. Dirks D. (1996). Report of the eriksholm workshop on auditory deprivation and acclimatization. *Ear Hear.* 17(Suppl. 3) 87S–98S. 10.1097/00003446-199617031-00009 8807279

[B6] Brehmer Y. Westerberg H. Backman L. (2012). Working-memory training in younger and older adults: training gains, transfer, and maintenance. *Front. Hum. Neurosci.* 6:63 10.3389/fnhum.2012.00063 PMC3313479 22470330

[B7] Burk M. H. Humes L. E. Amos N. E. Strauser L. E. (2006). Effect of training on word-recognition performance in noise for young normal-hearing and older hearing-impaired listeners. *Ear Hear.* 27 263–278. 10.1097/01.aud.0000215980.21158.a2 16672795

[B8] Chan R. C. K. Shum D. Toulopoulou T. Chen E. Y. H. (2008). Assessment of executive functions: review of instruments and identification of critical issues. *Arch. Clin. Neuropsychol.* 23 201–216. 10.1016/j.acn.2007.08.010 18096360

[B9] Cohen J. (1988). *Statistical Power Analysis for the Behavioral Sciences*, 2nd Edn. Hillsdale, NJ: Lawrence Earlbaum Associates.

[B10] de Boer J. Thornton A. R. D. (2008). Neural correlates of perceptual learning in the auditory brainstem: efferent activity predicts and reflects improvement at a speech-in-noise discrimination task. *J. Neurosci.* 28 4929–4937. 10.1523/JNEUROSCI.0902-08.2008 18463246 PMC6670751

[B11] Dunning D. L. Holmes J. (2014). Does working memory training promote the use of strategies on untrained working memory tasks? *Mem. Cognit.* 42 854–862. 10.3758/s13421-014-0410-5 PMC4110412 24748348

[B12] Ferguson M. A., Henshaw H. Computer and internet interventions to optimise listening and learning for people with hearing loss: accessibility, use and adherence.. *Am. J. Audiol.*.

[B13] Ferguson M. A. Henshaw H. Clark D. Moore D. (2014). Benefits of phoneme discrimination training in a randomized controlled trial of 50–74 year olds with mild hearing loss. *Ear Hear.* 35 e110–e121. 10.1097/AUD.0000000000000020 24752284 PMC4072445

[B14] Foo C. Rudner M. Ronnberg J. Lunner T. (2007). Recognition of speech in noise with new hearing instrument compression release settings requires explicit cognitive storage and processing capacity. *J. Am. Acad. Audiol.* 18 618–631. 10.3766/jaaa.18.7.8 18236648

[B15] Fu Q. J. Galvin J. Wang X. Nogaki G. (2004). Effects of auditory training on adult cochlear implant patients: a preliminary report. *Cochlear Implants Int.* 5 84–90. 10.1179/cim.2004.5.Supplement-1.84 18792249

[B16] Gatehouse S. (1999). Glasgow hearing aid benefit profile: derivation and validation of client-centred outcome measures for hearing aid services. *J. Am. Acad. Audiol.* 10 80–103.

[B17] Gatehouse S. Naylor G. Elberling C. (2003). Benefits from hearing aids in relation to the interaction between the user and the environment. *Int. J. Audiol.* 42 S77–S85. 10.3109/14992020309074627 12918613

[B18] Gatehouse S. Noble W. (2004). The Speech, Spatial and Qualities of Hearing Scale (SSQ). *Int. J. Audiol.* 43 85–99. 10.1080/14992020400050014 15035561 PMC5593096

[B19] Gordon-Salant S. (2014). “Aging, hearing loss, and speech recognition: stop shouting, i can’t understand you,” in *Perspectives on Auditory Research*, eds Popper A. N. Fay R. R. (New York, NY: Springer), 211–228.

[B20] Hazan V. Messaoud-Galusi S. Rosen S. Nouwens S. Shakespeare B. (2009). Speech perception abilities of adults with dyslexia: is there any evidence for a true deficit? *J. Speech Lang. Hear. Res.* 52 1510–1529. 10.1044/1092-4388(2009/08-0220) 19635940

[B21] Heinrich A. Henshaw H. Ferguson M. A. (2015). The relationship of speech intelligibility with hearing sensitivity, cognition, and perceived hearing difficulties varies for different speech perception tests. *Front. Psychol.* 6:782 10.3389/fpsyg.2015.00782 PMC4468362 26136699

[B22] Henshaw H. Ferguson M. A. (2013a). Efficacy of individual computer-based auditory training for people with hearing loss: a systematic review of the evidence. *PLoS ONE* 8:e62836 10.1371/journal.pone.0062836 PMC3651281 23675431

[B23] Henshaw H. Ferguson M. A. (2013b). Working memory training for adult hearing aid users: study protocol for a double-blind randomized active controlled trial. *Trials* 14:417. 10.1186/1745-6215-14-417 PMC4235173 24304745

[B24] Henshaw H. Ferguson M. A. (2014). “Assessing the benefits of auditory training to real-world listening: identifying appropriate and sensitive outcomes,” in *Proceedings of ISAAR 2013: Auditory Plasticity – Listening with the Brain. 4th symposium on Auditory and Audiological Research*, eds Dau T. Santurette S. Dalsgaard J. C. Trangjaerg L. Andersen T. Poulsen T. (Nyborg: The Danavox Jubilee Foundation).

[B26] Hopkins K. Moore B. C. (2011). The effects of age and cochlear hearing loss on temporal fine structure sensitivity, frequency selectivity, and speech reception in noise. *J. Acoust. Soc. Am.* 130 334–349. 10.1121/1.3585848 21786903

[B27] Howard C. S. Munro K. J. Plack C. J. (2010). Listening effort at signal-to-noise ratios that are typical of the school classroom. *Int. J. Audiol.* 49 928–932. 10.3109/14992027.2010.520036 21047295

[B28] Humes L. E. Dubno J. R. (2010). “Factors affecting speech understanding in older adults,” in *The Aging Auditory System*, eds Gordon-Salant S. Frisna R. D. Popper A. N. Fay R. R. (New York, NY: Springer), 211–257.

[B29] Humes L. E. Kidd G. R. Lentz J. J. (2013). Auditory and cognitive factors underlying individual differences in aided speech-understanding among older adults. *Front. Syst. Neurosci.* 7:55 10.3389/fnsys.2013.00055 PMC3787592 24098273

[B30] Johnson E. E. Dillon H. (2011). A comparison of gain for adults from generic hearing aid prescriptive methods: impacts on predicted loudness, frequency bandwidth, and speech intelligibility. *J. Am. Acad. Audiol.* 22 441–459. 10.3766/jaaa.22.7.5 21993050

[B31] Kiessling J. Pichora-Fuller M. K. Gatehouse S. Stephens D. Arlinger S. Chisolm T. (2003). Candidature for and delivery of audiological services: special needs of older people. *Int. J. Audiol.* 42 S92–S101. 10.3109/14992020309074650 12918635

[B32] Kochkin S. (2010). MarkeTrak VIII: consumer satisfaction with hearing aids is slowly increasing. *Hear. J.* 63 19–20. 10.1097/01.HJ.0000366912.40173.76

[B33] Kronenberger W. G. Pisoni D. B. Henning S. C. Colson B. G. Hazzard L. M. (2011). Working memory training for children with cochlear implants: a pilot study. *J. Speech Lang. Hear. Res.* 54 1182–1196. 10.1044/1092-4388(2010/10-0119) 21173394 PMC3293211

[B34] Lunner T. (2003). Cognitive function in relation to hearing aid use. *Int. J. Audiol.* 42 S49–S58. 10.3109/14992020309074624 12918610

[B35] Lunner T. Sundewall-Thorén E. (2007). Interactions between cognition, compression, and listening conditions: effects on speech-in-noise performance in a two-channel hearing aid. *J. Am. Acad. Audiol.* 18 604–617. 10.3766/jaaa.18.7.7 18236647

[B36] Mcarthur G. (2007). Test–retest effects in treatment studies of reading disability: the devil is in the detail. *Dyslexia* 13 240–252. 10.1002/dys.355 17948880

[B37] Melby-Lervag M. Hulme C. (2013). Is working memory training effctive? A meta- analytic review. *Dev. Psychol.* 49 270–291. 10.1037/a0028228 22612437

[B38] Millward K. Moore D. R. Sohoglu E. Amitay S. (2011). Training speech-in-noise perception in mainstream school children. *Int. J. Pediatr. Otorhinolaryngol.* 75 1408–1417. 10.1016/j.ijporl.2011.08.003 21889805

[B39] Moore D. R. Edmondson-Jones M. Dawes P. Fortnum H. Mccormack A. Pierzycki R. H. (2014). Relation between speech-in-noise threshold, hearing loss and cognition from 40–69 years of age. *PLoS ONE* 9:e107720 10.1371/journal.pone.0107720 PMC4168235 25229622

[B40] Ng E. H. Classon E. Larsby B. Arlinger S. Lunner T. Rudner M. (2014). Dynamic relation between working memory capacity and speech recognition in noise during the first 6 months of hearing aid use. *Trends Hear.* 18 2331216514558688 10.1177/2331216514558688 PMC4271770 25421088

[B41] Oba S. I. Fu Q. Galvin J. J. (2011). Digit training in noise can improve cochlear implant users’ speech understanding in noise. *Ear Hear.* 32 573–581. 10.1097/AUD.0b013e31820fc821 21389857 PMC3129451

[B42] Perkins D. N. Salomon G. (1992). “Transfer of learning,” in *International Encyclopedia of Education*, 2nd Edn eds Husén T. Postlethwaite T. (Oxford: Pergamon Press).

[B43] Pichora-Fuller M. K. Levitt H. (2012). Speech comprehension training and auditory and cognitive processing in older adults. *Am. J. Audiol.* 21 351–357. 10.1044/1059-0889(2012/12-0025) 23233521

[B44] Pichora-Fuller M. K. Singh G. (2006). Effects of age on auditory and cognitive processing: implications for hearing aid fitting and audiologic rehabilitation. *Trends Amplif.* 10 29–59. 10.1177/108471380601000103 16528429 PMC4111543

[B45] Robertson I. H. Ward T. Ridgeway V. Nimmo-Smith I. (1996). The structure of normal human attention: The Test of Everyday Attention. *J. Int. Neuropsychol. Soc.* 2 525–534. 10.1017/S1355617700001697 9375156

[B46] Ronnberg J. Lunner T. Zekveld A. Sorqvist P. Danielsson H. Lyxell B. (2013). The Ease of Language Understanding (ELU) model: theoretical, empirical, and clinical advances. *Front. Syst. Neurosci.* 7:31 10.3389/fnsys.2013.00031 PMC3710434 23874273

[B47] Rudner M. Rönnberg J. Lunner T. (2011). Working memory supports listening in noise for persons with hearing impairment. *J. Am. Acad. Audiol.* 22 156–167. 10.3766/jaaa.22.3.4 21545768

[B48] Sarampalis A. Kalluri S. Edwards B. Hafter E. (2009). Objective measures of listening effort: effects of background noise and noise reduction. *J. Speech Lang. Hear. Res.* 52 1230–1240. 10.1044/1092-4388(2009/08-0111) 19380604

[B49] Schow R. Nerbonne M. (2006). *Introduction to Audiologic Rehabilitation*. Boston, MA: Pearson Education.

[B50] Shinn-Cunningham B. G. (2008). Object-based auditory and visual attention. *Trends Cogn. Sci.* 12 182–186. 10.1016/j.tics.2008.02.003 18396091 PMC2699558

[B51] Shinn-Cunningham B. G. Best V. (2008). Selective attention in normal and impaired hearing. *Trends Amplif.* 12 283–299. 10.1177/1084713808325306 18974202 PMC2700845

[B52] Smith G. E. Housen P. Yaffe K. Ruff R. Kennison R. F. Mahncke H. W. (2009). A cognitive training program based on principles of brain plasticity: results from the Improvement in Memory with Plasticity-based Adaptive Cognitive Training (IMPACT) study. *J. Am. Geriatr. Soc.* 57 594–603. 10.1111/j.1532-5415.2008.02167.x 19220558 PMC4169294

[B53] Smits C. Kapteyn T. S. Houtgast T. (2004). Development and validation of an automatic speech-in-noise screening test by telephone. *Int. J. Audiol.* 43 15–28. 10.1080/14992020400050004 14974624

[B54] Song J. H. Skoe E. Banai K. Kraus N. (2011). Training to improve hearing speech in noise: biological mechanisms. *Cereb. Cortex* 22 1180–1190. 10.1093/cercor/bhr196 21799207 PMC3450924

[B55] Stacey P. C. Raine C. H. O’donoghue G. M. Tapper L. Twomey T. Summerfield A. Q. (2010). Effectiveness of computer-based auditory training for adult users of cochlear implants. *Int. J. Audiol.* 49 347–356. 10.3109/14992020903397838 20380610

[B56] Stecker G. C. Bowman G. A. Yund E. W. Herron T. J. Roup C. M. Woods D. L. (2006). Perceptual training improves syllable identification in new and experienced hearing aid users. *J. Rehabil. Res. Dev.* 43 537–551. 10.1682/JRRD.2005.11.0171 17123192

[B57] Sweetow R. W. Sabes J. H. (2006). The need for and development of an Adaptive Listening and Communication Enhancement (LACE) Program. *J. Am. Acad. Audiol.* 17 538–558. 10.3766/jaaa.17.8.2 16999250

[B58] Thompson T. W. Waskom M. L. Garel K. A. Cardenas-Iniguez C. Reynolds G. O. Winter R. (2013). Failure of working memory training to enhance cognition or intelligence. *PLoS ONE* 8:e63614 10.1371/journal.pone.0063614 PMC3661602 23717453

[B59] Tremblay K. (2007). Training-related changes in the brain: evidence from human auditory-evoked potentials. *Semin. Hear.* 28 120–132. 10.1055/s-2007-973438

[B60] Tyler R. S. Witt S. A. Dunn C. C. Wang W. (2010). Initial development of a spatially separated speech-in-noise and localization training program. *J. Am. Acad. Audiol.* 21 390–403. 10.3766/jaaa.21.6.4 20701836 PMC2947843

[B61] Vermiglio A. J. Soli S. D. Freed D. J. Fisher L. M. (2012). The relationship between high-frequency pure-tone hearing loss, hearing in noise test (HINT) thresholds, and the articulation index. *J. Am. Acad. Audiol.* 23 779–788. 10.3766/jaaa.23.10.4 23169195

[B62] Wechsler D. (1997). *Wechsler Adult Intelligence Scale*, 3rd Edn. San Antonio, TX: The Psychological Corporation.

[B63] Wright B. A. Zhang Y. (2009). A review of the generalization of auditory learning. *Philos. Trans. R. Soc. B Biol. Sci.* 364 301–311. 10.1098/rstb.2008.0262 PMC2674478 18977731

